# Biased competition through variations in amplitude of *γ*-oscillations

**DOI:** 10.1007/s10827-007-0066-2

**Published:** 2008-02-22

**Authors:** Magteld Zeitler, Pascal Fries, Stan Gielen

**Affiliations:** 1 Department of Biophysics, Institute for Neuroscience, Radboud University Nijmegen, Geert Grooteplein 21, 6525 EZ Nijmegen, The Netherlands; 2 F.C. Donders Centre for Cognitive Neuroimaging, Radboud University Nijmegen, Kapittelweg 29, 6525 EN Nijmegen, The Netherlands

**Keywords:** Selective attention, Stimulus competition, Coherence, Temporal correlated spike input

## Abstract

Experiments in visual cortex have shown that the firing rate of a neuron in response to the simultaneous presentation of a preferred and non-preferred stimulus within the receptive field is intermediate between that for the two stimuli alone (stimulus competition). Attention directed to one of the stimuli drives the response towards the response induced by the attended stimulus alone (selective attention). This study shows that a simple feedforward model with fixed synaptic conductance values can reproduce these two phenomena using synchronization in the gamma-frequency range to increase the effective synaptic gain for the responses to the attended stimulus. The performance of the model is robust to changes in the parameter values. The model predicts that the phase locking between presynaptic input and output spikes increases with attention.

## Introduction

Our retinas are constantly stimulated by an overwhelming amount of information and the brain faces the task of reducing a potentially overloading amount of information into a manageable flow that reflects both the current needs of the organism and the external demands placed on it. In order to solve this problem, the brain uses a strategy to select the relevant information and to suppress information which is not relevant. The focus on and selection of relevant information is referred to as “attention”. If just one single stimulus falls within the receptive field of a neuron, this stimulus can be attended or not, and in the latter case a stimulus outside the receptive field may be attended. Since higher cortical areas have large receptive fields (Smith et al. 2002), it is quite common that two (or even more) stimuli fall within the receptive field of a neuron. In that case one of them can be attended (selective attention) or none of them. In order to understand the neuronal substrate of attention, many single-unit studies in visual cortex have investigated how attended and unattended stimuli are encoded in the firing rate of neurons.

Neural correlates of attention have been studied using single-unit recordings in areas V1, V2, V4 and V5/MT in primate visual cortex. Several studies have shown that attention increases a neuron’s firing rate in response to a single stimulus in its receptive field (Treue and Maunsell 1999; Luck et al. 1997; Reynolds et al. 1999; McAdam and Maunsell 1999; Fries et al. 2001). When two stimuli are presented in the receptive field of the neuron, the firing rate lies between the firing rates elicited by each of the stimuli presented alone (Moran and Desimone 1985; Treue and Maunsell 1996, 1999; Luck et al. 1997; Chelazzi et al. 1998, 2001; Reynolds et al. 1999; Reynolds and Desimone 2003). This phenomenon is called stimulus competition, since populations of input neurons, encoding different stimuli, are thought to compete with one another to generate neuronal responses intermediate between the responses to the individual stimuli. When attention is directed to the neuron’s preferred stimulus, the neuron’s firing rate increases, whereas attention to the non-preferred stimulus decreases the firing rate (Chelazzi et al. 1998; Reynolds et al. 1999).

Several models have been proposed to reproduce these experimental observations regarding stimulus competition and selective attention. Reynolds et al. (1999) could explain their experimental results by assuming that the synaptic weights of an input representing one of the two stimuli increase five-fold when attention is directed towards that stimulus. However, it is not clear how synaptic efficacies could change five-fold at the time scale of attentional shifts.

Most approaches to come up with an explanation for stimulus competition and selective attention have focused on the effects of attention on the firing rate of neurons (see e.g. Tiesinga 2005; Deco and Rolls 2005; Buia and Tiesinga 2006; Mishra et al. 2006). In addition to firing rate, several studies have provided convincing evidence that selective attention also increases rhythmic synchronization among selected neuronal signals (Kreiter and Singer 1996; Fries et al. 2001; Schoffelen et al. 2005; Taylor et al. 2005; Womelsdorf et al. 2006). Several groups have published a model for neural implementation of attentional processes that attributes a possible role to the neuronal oscillatory activity in stimulus competition and/or selective attention (Tiesinga 2005; Buia and Tiesinga 2006; Mishra et al. 2006). Mishra et al. (2006) used gamma range correlations in the feedforward inhibitory inputs to the V4 neuron which are out of phase with the gamma band correlations within the excitatory input corresponding to the attended stimulus. Tiesinga (2005) used two asynchronous excitatory input populations and two stimulus-driven inhibitory input populations, which send 40 Hz spike volleys with some temporal dispersion to a V4 model neuron. In that study attention is modelled by changing the temporal dispersion or the relative phase between the volleys coming from the two inhibitory populations. Tiesinga (2005) used the crosscorrelation function as a measure for the synchronization between the responses of two V4 neurons. Since he did this only for the condition that two stimuli are presented in the same receptive field, it is difficult to compare the result with the experimental results of one stimulus within and one outside the receptive field of a neuron as measured by Fries et al. (2001). Another measure for the synchronization between two signals is the coherence function. We will use the coherence function as a measure in the frequency domain for the synchronization between the input and output of the excitatory neuron in our model for different conditions.

Since it is well known that the excitatory input in visual cortex from V1 to V2 and from V2 to V4 contains gamma frequency oscillations (Eckhorn et al. 1993; Frien et al. 1994; Maldonado et al. 2000), we have explored the possible role of gamma frequency oscillatory input in stimulus competition and selective attention. We tried to reproduce the experimental observations by a simple feedforward model. Therefore, the aim of the present study was to explore whether a simple feedforward model could explain the phenomenon of stimulus competition with a role for synchronous modulation of stimulus-related activity to implement the attentional bias. Our results show that a feedforward model, very similar to the gain modulation model of Reynolds et al. (1999) but with fixed synaptic weights, can explain stimulus competition. Assuming that attention is implemented by increased synchronization of multi-unit spike activity, the model can reproduce the results by Chelazzi et al. (1998) and Reynolds et al. (1999) on stimulus competition and selective attention. Although this model has a feedforward architecture, the underlying mechanism for changes in attention-related modulations of synchronous activity is not specified, this requires a role for some top–down feedback mechanism capable of enhancing synchrony.

## Methods and theory

We will start this section with a description of our model and the input signals to the model. In the second part of this section we will describe the methods to calculate the coherence, the phase coherence and the phase locking value between synaptic input and spike output.

### Model

**Fig. 1:**
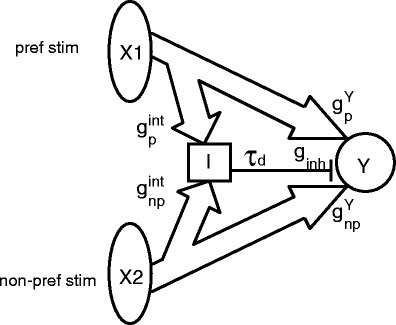
Schematic overview of the simple feedforward model. A preferred and a non-preferred stimulus are represented by spike trains, coming from two populations (*X*1 and *X*2) of 80 Poisson model neurons, each. These two populations project to a population of 40 Hodgkin–Huxley type interneurons (*I*) and to the Hodgkin–Huxley type output neuron *Y*. Each population receives its own time-dependent rate defined in Eq. (1). Therefore, the spike trains within a population are correlated with each other, but not with spike trains in the other population. The two population activities are statistically the same as long as they are both unattended or both attended. The difference between responses to preferred and non-preferred stimulus is determined by the different synaptic conductances. Population *X*2 (non-preferred stimulus) has stronger projections to the interneurons *I* and weaker to the output neuron *Y* than population *X*1 (preferred stimulus) ($g_{\rm np}^{\rm int} \: > \: g_{\rm p}^{\rm int}$ and $g_{\rm np}^Y \: < \: g_{\rm p}^Y$). Spikes, generated by the interneurons arrive after a short delay *τ*_*d*_ of 2 ms at neuron *Y*. In addition both HH-like neurons (*I* and *Y*) receive background noise, represented by conductance injections in the soma

Figure 1 shows the feedforward network, that we propose to explain stimulus-competition and selective attention. The output neuron *Y* receives excitatory spike-trains from two populations (*X*1 and *X*2) with 80 Poisson neurons each and also receives inhibitory input from a population of 40 inhibitory neurons, for brevity called interneurons, I. In this study *X*1 and *X*2 represent the population of neurons encoding the preferred and non-preferred stimulus, respectively. With two populations of 80 excitatory neurons and a population of 40 inhibitory neurons projecting to the output neuron, the ratio of excitatory versus inhibitory synapses is 80 vs. 20% in agreement with experimental observations (Beaulieu et al. 1992). The two excitatory populations of neurons also project to the interneuron population. There is a small time delay *τ*_*d*_ of 2 ms between the spike times of the interneurons and the arrival times of these spikes at neuron *Y*. The interneurons and the output neuron *Y* have been implemented in NEURON, as Hodgkin–Huxley type neurons (see below).

### Stimulus-related input signals

The outputs from *X*1 and *X*2 are Poisson trains of spikes with a time-dependent rate *r* ^*i*^(*t*):

1 $$ r^i(t) = r + A^i_{m} \eta^i(t) $$

with *i*  *ε* {1,2}, *r* the constant rate, *η* ^*i*^ bandpass filtered Gaussian white noise with 3dB points at 45 and 55 Hz, a quality factor Q of 5, zero mean and a variance of one, and with $A^i_m$ the modulation amplitude of the Gaussian white noise (GWN) for population *i*. When the modulation amplitudes $A_m^i$ are the same for the non-preferred and the preferred stimulus, the spike trains encoding the non-preferred and the preferred stimulus are statistically identical. The different responses of the output neuron to the two stimulus inputs are due to the differences in synaptic conductances of the projections of the two populations of Poisson neurons to the inhibitory neurons and output neuron (will be explained later). Since we are not aware of any hard physiological data about these synaptic conductances in the literature, the different projections of the preferred and non-preferred stimulus to the interneurons and to the output neuron are an assumption of the model.

Several studies have shown that attention to a visual stimulus results in increased coherence between the local field potential and the activity of neurons, especially in the *γ*-band range  (Fries et al. 2001; Womelsdorf et al. 2006). In the visual system *γ*-band oscillations have been reported at frequencies in the range 40–80 Hz. Based on these findings we postulate that selective attention to a sensory stimulus is implemented as an increased amplitude *A*_*m*_ for the neuronal activity encoding that stimulus. For the simulations of the responses of the output neuron *Y* to various input signals we used a time duration *T* of 8 092 s and time step *dt* of 0.1 ms. The spike trains of the two Poisson populations *X*_1_ and *X*_2_ were modulated by a constant mean rate *r* = 20 and with a modulation amplitude *A*_*m*_ = 6 for a non-attended stimulus and *A*_*m*_ = 8 for an attended stimulus (see Eq. (1)). If no input is presented to *X*_1_ or *X*_2_, *r* = 3 and *A*_*m*_ = 0. In order to explore the role of the modulation amplitude on the results of this study, some simulations used a modulation amplitude of 12 and 16 for the unattended and attended stimulus, respectively.

### Geometry and properties of the HH-type interneurons and output neuron

The interneurons and output neuron *Y* were implemented in the NEURON simulation environment (Hines and Carnevale 1997) as single-compartment Hodgkin–Huxley type neurons with an area of 34,636 $\upmu$m^2^, in agreement with Destexhe et al. (2001). The inhibitory interneurons contain two sets of 80 synapses, the output neuron *Y* has 40 inhibitory and two sets of 80 excitatory synapses. The synaptic conductivity *g* is modelled by the default alpha function in NEURON. In this study most results were obtained for modulation amplitude *A*_*m*_ values of 6 or 8. In that case the excitatory synapses from the populations *X*_1_ and *X*_2_ onto the interneurons have a maximum conductance of $g^{\rm int}_{\rm np}$ = 0.84 nS and $g^{\rm int}_{\rm p}$ = 0.55 nS for the non-preferred and preferred stimulus input, respectively and a time constant *τ*_*e*_ = 2 ms. For the excitatory synapses onto the output neuron *Y*, the following values are taken: $g^{Y}_{\rm np}$ = 1.52 nS, $g^{Y}_{\rm p}$ = 1.71 nS, *τ*_*e*_ = 2 ms. For the synapses from the inhibiting interneurons to the output neuron *Y* we had *g*_inh_ = 4.5 nS and *τ*_*i*_ = 5 ms. For modulation amplitudes *A*_*m*_ with values of 12 (‘no attention’) and 16 (‘with attention’) the synaptic conductance values were $g^{\rm int}_{\rm np}$ = 0.84 nS, $g^{\rm int}_{\rm p}$ = 0.55 nS, $g^{Y}_{\rm np}$ = 1.52 nS, $g^{Y}_{\rm p}$ = 1.71 nS and *g*_inh_ = 3.8 nS. With these values for *A*_*m*_ = 12 the output neuron in our model generates, in agreement with experimental data of Reynolds et al. (1999), a firing rate (*f*_p_) of about 20 sp/s in response to the ‘preferred’ stimulus condition and a firing rate (*f*_np_) of about 10 sp/s in response to the ‘non-preferred’ stimulus.

The somata of the Hodgkin–Huxley type neurons have passive and active cell properties. The passive properties are the leak reversal potential (− 80 mV), leak conductance (4.52   10^ − 5^ S/cm^2^) and membrane capacitance (1 $\upmu$F/cm^2^). The active properties refer to the voltage-dependent Na^ + ^ current and the “delayed-rectifier” K^ + ^ current. The parameter values for the voltage-dependent Na^ + ^ and K^ + ^ currents were as described by Traub and Miles (1991) (see Appendix 1).

The synaptic background activity of the Hodgkin–Huxley-like neurons (interneurons and output neuron) was approximated by conductance injections in the soma as described in Destexhe et al. (2001) (see Appendix 1). In agreement with Destexhe et al. (2001), we used the following parameter values for the output neuron: the reversal potentials of the excitatory and inhibitory inputs *E*_*e*_ = 0 mV, *E*_*i*_ = − 75 mV, the average conductances *g*_*eo*_ = 12.1 nS, *g*_*i*0_ = 57.3 nS and the time constants *τ*_*e*_ = 2.73 ms, *τ*_*i*_ = 10.49 ms. The standard deviations of the conductances corresponding to the background activity of output neuron *Y* are given by *σ*_*e*_ = 3.0 nS and *σ*_*i*_ = 6.0 nS. For the interneurons the average conductances and the standard deviations of these conductances are 50% of the corresponding values of the output neuron.

In order to understand the responses of the interneurons, it is helpful to appreciate the relative size of the synaptic currents due to the background noise and due to stimulus related inputs. These synaptic currents due to the spike input are rough estimates, since the precise relation between spike input and synaptic current depends on the membrane potential of the neuron, and thereby also depends on other synaptic inputs that affect the membrane potential. Assuming that the mean membrane potential is near − 55 mV (i.e. halfway between the membrane potential at rest near − 75 mV and the threshold for firing) the mean current due to background activity for the interneurons is about 60% of the total excitatory input current. The remaining 40% comes from the mean excitatory input related to the preferred stimulus (16%) and to the non-preferred stimulus (24%). For the output neuron, the inhibitory stimulus related input is about 20% of the current due to the background activity, whereas the excitatory stimulus related input is about 85% of the background current. More details on these relative contributions and their effect on the relation between mean input current and firing rate is provided in Appendix 2.

### Coherence estimate

One of the predictions that flows from our hypothesis (see Section 1) is that the output spike train is more coherent to the “attended” input spike train than to the “ignored” input spike train. To quantify this, we will use the coherence function, in addition to firing rate to investigate the effect of attention on the spike output of neuron *Y*. In order to distinguish between the effect of the non-preferred and the preferred stimulus on the spike output, the non-preferred and preferred stimulus are statistically uncorrelated ($<\eta_i^{(t)}\eta_j^{(t)} > = \delta_{ij}$). This is in agreement with Gray et al. (1989) and Kreiter and Singer (1996) who reported that correlations between neuron population activities encoding different stimuli are absent.

The coherence function *γ*(*ω*) reflects how much of the variations in the output *y* can be attributed to a linear filtering of the input signal *x*. The coherence function *γ*(*ω*) is defined by:

2 $$ \mid \gamma(\omega) \mid = \frac{\mid C_{xy}(\omega)\mid} {\sqrt{\mid C_{xx}(\omega) \mid}\sqrt{\mid C_{yy}(\omega) \mid}} $$

with *C*_*xy*_(*ω*) the Fourier transform of the cross covariance function (Marmarelis and Marmarelis 1978). The coherence takes values in the range between 0 (input and output are uncorrelated) and 1 (the output is equal to the input after convolution by a linear system). Since the neuron itself is not a linear system, the coherence between the bandpass filtered Gaussian white noise input of one of the two Poisson populations and the spike output of neuron *Y* will not reach the upper limit of one.

To estimate the coherence and its variance, we used the multi-taper method (Thomson 1982; Mitra and Pesaran 1999). The key idea behind the multi-taper method is that a physiological signal does not have discontinuities in the frequency spectrum and that the variance in the estimate of a signal can be reduced by smoothing in the frequency domain. The multi-taper method minimizes bias and variance of the estimate by using multiple orthonormal data tapers. We have used sine-tapers as described in Zeitler et al. (2006) with length *N* = 1.024 s and bandwidth *W* = 2.9 Hz. Since the number of tapers to be used is *K* = 2*NW* − 1 tapers, the values for *N* and *W* used in this study gave *K* = 5. The binwidth in the frequency domain is the Rayleigh frequency *f*_*r*_ = 1/*T* = 1/(*nfft*/*f*_*s*_) = 0.98 Hz, with sampling frequency *f*_*s*_ (1,000 Hz) and where nfft (1,024) is the number of data points in the FFT . The input and output signals were both segmented in *T*/*N* non-overlapping time segments of 1,024 ms, with *T* the duration of the simulation.

### Phase locking

A high value of the regular coherence Eq. (2) implies a strong relation between both amplitude and phase of input and output. Previous studies have shown that pairs of neuronal responses can undergo variations in relative amplitude even in the presence of tight phase coupling (Tass et al. 1998; Lachaux et al. 1999). For this reason, the phase coherence has been introduced, which only considers the variability in relative phase between two signals *s*1 and *s*2. In this study, the phase coherence is calculated by segmentation of the two signals *s*1(*t*) and *s*2(*t*), both segmented in *T*/*N* non-overlapping time segments of 1,024 ms. Each segment of the signal *s*1 and the corresponding segment of the second signal *s*2 form a pair. The phase difference *Δϕ*(*f*) at frequency *f* for each pair is given by:

3 $$ \exp( i\Delta \varphi (f)) = \frac{S1(f) S2^*(f)}{| S1(f) S2(f)| } $$

where ^*^ refers to complex conjugate.

**Fig. 2:**
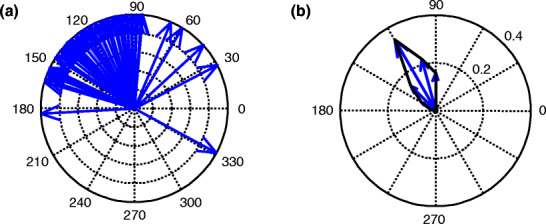
Illustration of phase-coherence analysis between stimulus and response. (**a**) Shows a polar plot of the phase differences for 150 pairs of stimulus and response. In this example, the stimulus and its response have a preferred phase difference in the range between 90 and 150°. (**b**) Shows data in (**a**) in a polar plot. The length of the arrows show the fraction of phase differences falling in the corresponding phase bin

Figure 2(a) shows a typical polar plot of the phase differences between 150 stimulus–response pairs for the neuron model in Fig. 1. The full range of 360° was subdivided into 24 bins of 15° [15(*j* − 1), 15*j*] for *j* *ε* {1,..,24}. The number of phase differences falling into a bin, divided by the total number of phase differences in the unit circle, is the fraction of stimulus–response pairs with a phase difference in that bin. For each of the twenty bins, this fraction is represented by the length of the arrow, drawn in the middle of each bin [see Fig. 2(b)]. All fractions are connected by a line. When stimulus and response are not phase locked at all, the phase differences will be distributed uniformly over 360°. Complete phase locking with phase difference *Φ* corresponds to an arrow of unit length pointing in the direction *Φ*.

Lachaux et al. (1999) introduced a method to quantify the degree of phase-locking between two signals. The phase locking value (*PLV*) between the two periodically repeated signals measures the inter-trial variability of the phase difference between these two signals. In our simulations, we average the phase relation over all stimulus–response pairs of the *M* = *T*/*N* time segments:

4 $$ PLV (f) = \left| \frac{1}{M} \sum_{m=1}^M \exp(i~\Delta\varphi_m(f))\right| $$

This phase locking value measures the average variability of the phase difference and takes values between 0 (complete lack of phase-locking) and 1 (completely phased locked).

The phase locking value is a function of frequency. In order to reduce the variance of the phase locking value, we used the multi-taper method, with *K* = 5 sine-tapers to reduce the variance of the spectra *S*1(*f*) and *S*2(*f*) in Eq. (3). Since the phase locking value was very similar for all frequencies near 50 Hz, we determined the phase locking value for *f* = 50 Hz as this gave the best signal-to-noise ratio for the 50 Hz bandpass filtered Gaussian white noise input.

## Results

In this section the simulation results will be described for the firing rate of the output neuron *Y* (Section  3.2) and the coherences between the spikes of the output neuron and each of the stimulus-related inputs to the populations of Poisson neurons (Section 3.3). We will conclude this section with the phase locking results (Section 3.4).

### Input–output relation of an interneuron

**Fig. 3:**
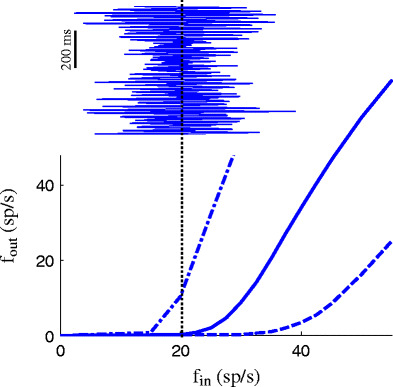
Relation between constant firing rate of neural activity representing the non-preferred and preferred stimulus and firing rate at the output of one interneuron for “non-preferred stimulus only” (*solid line*), “preferred stimulus only” (*dashed line*) and for “both stimuli” (*dashed-dotted line*). For the condition “non-preferred stimulus only” (*solid line*), the input to the interneuron has two components. One component represents the non-preferred stimulus by 80 Poisson spike series, each with a constant firing rate *f*_in_. The other component represents the activity of 3 spikes/s in the population encoding the absence of the preferred stimulus. The *dashed line* shows the output of the interneuron for the preferred stimulus only. The *dashed-dotted line* shows the output of the interneuron to both stimuli, each represented by 80 Poisson spike series with a constant firing rate *f*_in_. The *inset* shows the population activity of GWN-modulated Poison spike series, according to Eq. (1) with *A*_*m*_ = 6

The interneuron plays a crucial role to explain stimulus competition (Fig. 3). If an interneuron receives input of the preferred stimulus with firing rate *f*_in_ (this implies that all 80 neurons encoding the preferred stimulus have a constant firing rate of *f*_in_ and the 80 neurons encoding the (absent) non-preferred stimulus have a firing rate of 3 spikes/s) the interneuron starts to respond at relatively high input firing rates (dashed line). Since the synaptic projections to the interneuron of the neural activity encoding the non-preferred stimulus is stronger than for the neural activity related to the preferred stimulus, the relation shifts to the left for the non-preferred stimulus only (solid line, lower threshold for firing). The inset shows the population activity of the Gaussian white noise (GWN) modulated Poisson spike input. This explains why the mean firing rate of the interneuron increases with increasing amplitude of the GWN-modulated Poisson spike input. For the output neuron qualitatively simular relations hold for the excitatory stimulus-related input, except for the fact that the output neuron responds better to the preferred stimulus alone, than to the non-preferred stimulus alone (solid and dashed lines interchanged).

### Simulation results for the firing rate

**Fig. 4:**
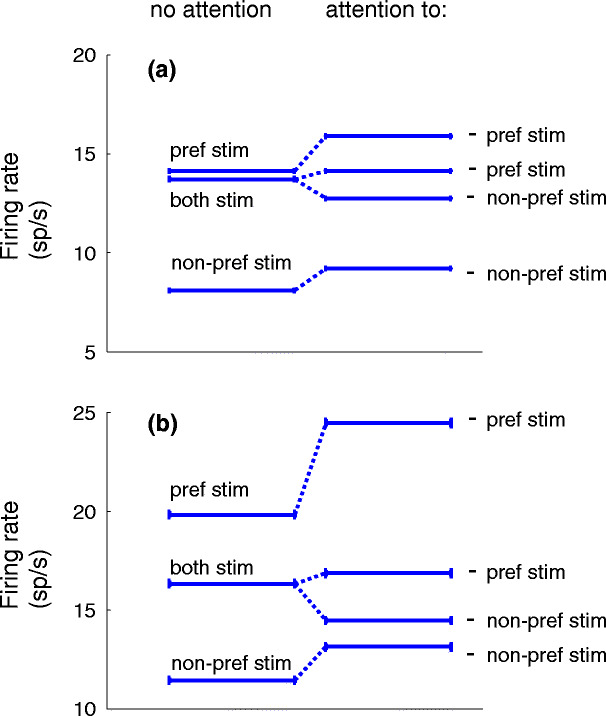
Mean firing rates of neuron *Y* for different stimulus combinations for the ‘with attention’ and ‘no attention’ condition. (**a**) Shows the results for small modulation amplitudes *A*_*m*_ (see text), (**b**) for two times larger modulation amplitudes. The *left side* shows the results for the ‘no attention’, the *right* for the ‘with attention’ condition. The firing rate for responses to the preferred stimulus and non-preferred stimulus increases when the preferred or non-preferred stimulus is attended. The firing rate for responses to both stimuli (*middle line on the left side*) is not the summation of the firing rates for each of the stimuli alone, but is in between. The *right side* shows that if both stimuli are presented, attention to the preferred stimulus increases the firing rate (*second line from the top*) and decreases the firing rate when non-preferred stimulus is attended (*second line from the bottom of the right side*). The following maximum conductance values are used for the simulations to obtain the firing rate results as shown in (**a**) and (**b**): $g^{\rm int}_{\rm np}$ = 0.84 nS, $g^{\rm int}_{\rm p}$ = 0.55 nS, $g^{Y}_{\rm np}$ = 1.52 nS, $g^{Y}_{\rm p}$ = 1.71 nS and *g*_inh_ = 4.50 nS for (**a**) and *g*_inh_ = 3.8 nS for (**b**)

Figure 4 shows the firing rate of the output neuron for various stimulus conditions. The upper panel (a) shows the results for small modulation amplitudes (*A*_*m*_ = 6 and 8), the lower panel (b) for larger modulation amplitudes (*A*_*m*_ = 12 and 16). The results at the left part of the figure show the results for the ‘no attention’ condition, the right part of the figure the results for the stimulus conditions with one stimulus attended. We will first discuss Fig. 4(a).

As explained in Section 2, the statistical properties of the spike series, representing the non-preferred and preferred stimulus with no attention, are identical. The different effectiveness of the non-preferred and preferred stimulus is mainly due to the different conductance of the excitatory synapses from *X*_1_ and *X*_2_ to the output neuron. Since $g_{\rm p}^{Y}$ (1.71 nS) > $g_{\rm np}^{Y}$ (1.52 nS) the direct excitatory projections of the population representing the preferred stimulus to the output neuron induce more action potentials in the output neuron than that of the population of neurons representing the non-preferred stimulus.

**Fig. 5:**
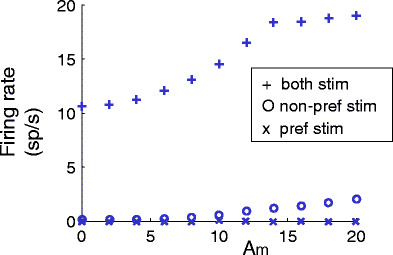
Input–output relationship of one interneuron. The response of an interneuron (firing rate in spikes/s) is shown as a function of the modulation amplitude *A*_*m*_ for the stimulus condition with the preferred (*x mark*) and non-preferred (*circle*) stimuli only, and for the condition with the preferred and non-preferred stimulus simultaneously (*plus symbol*). In the latter condition, the modulation amplitude was the same for both stimuli

The population activities representing the preferred (*X*_1_) and non-preferred stimulus (*X*_2_) also reach the output neuron via the interneurons. In case only one stimulus is offered, the interneurons have a low firing rate. This is shown in Fig. 5. For each stimulus separately, the induced firing rate of the interneurons is increasing as a function of the modulation amplitude *A*_*m*_. However, the firing rates in response to the preferred and non-preferred stimulus are rather small (range between 0 and 0.03 Sp/s and between 0 and 1.99 Sp/s for the preferred and non-preferred stimulus, respectively). Therefore, it is mainly the larger conductance of the excitatory synapses from population *X*_1_ to *Y* which explains the higher firing rate of the output neuron to the activity of population *X*_1_ (*f*_p_ = 14.15 sp/s, SD = 0.05 sp/s) than to the population activity *X*_2_ (*f*_np_ = 8.09 sp/s, SD = 0.04 sp/s), see left side of Fig. 4(a).

If the two neuronal populations, representing the activity of the preferred and non-preferred stimulus, would project to the output neuron only via excitatory synapses, one would expect a summation of firing rates when the preferred and non-preferred stimulus are presented simultaneously. However, we find stimulus competition in the responses of the output neuron, which is in agreement with experimental single-unit recordings (Reynolds et al. 1999), which most likely reflect the activity of excitatory neurons by their greater number and larger extracellular spikes. The interneurons play a crucial role in stimulus competition. This is illustrated in Fig. 5, which shows the response of an interneuron for the three conditions: ‘non-preferred stimulus only’, ‘preferred stimulus only’ and ‘preferred and non-preferred stimulus simultaneously’ as a function of the modulation amplitudes *A*_*m*_ of each stimulus. For all three conditions the response increases as a function of increasing *A*_*m*_ which shows that the interneurons are sensitive to correlated input. As mentioned before, the firing rate of the interneuron is very small when only one stimulus is presented. Therefore, the inhibition is small. When two stimuli are presented simultaneously the firing rate of the interneurons increases more than linearly due to the sigmoidal relation between synaptic input and firing rate of neurons in general. For the preferred and non-preferred stimulus alone the interneurons operate at the bottom of the sigmoidal relation, whereas the combined input of the preferred and non-preferred input shifts the firing rate to the steep phase of the sigmoidal relation, see also Fig. 11 in Appendix 2. So stimulus competition is caused by the activity of inhibitory interneurons, which generate a much higher firing rate when two stimuli are presented simultaneously compared to the condition that only one stimulus is presented. This higher response causes more inhibition for the target neuron and thus explains why the firing rate of the output neuron to both stimuli (*f*_both_ = 13.72 sp/s, SD = 0.05 sp/s) falls between the firing rates to the preferred and the non-preferred stimulus presented alone.

In summary, the responses to the preferred and non-preferred stimulus alone are mainly due to excitatory inputs and the difference in firing rates (*f*_np_ < *f*_p_) is caused by the different synaptic conductances. Competition (*f*_np_ < *f*_both_ < *f*_p_) is the net effect of the two direct excitatory inputs plus the inhibition via the interneurons, which are mainly actively if both stimuli are offered simultaneously.

Based on experimental observations that have revealed larger amplitudes of *γ*-range activity during attention (Fries et al. 2001; Womelsdorf et al. 2006; Taylor et al. 2005), attention to the preferred or non-preferred stimulus is implemented by a larger amplitude *A*_*m*_ of the band-pass filtered noise to the Poisson neurons. Increasing *A*_*m*_ leads to more spikes in the bursts of the population activity. Since the interneurons and output neuron receive a background synaptic input, they are sensitive to synchronous input (Martinez 2006; Higley and Contreras 2005). This explains the higher firing rate of the output neuron to the preferred ($f_{\rm p}^{\rm att}=$ 15.88 sp/s, SD = 0.05 sp/s) and non-preferred ($f_{\rm np}^{\rm att}=$ 9.18 sp/s, SD = 0.04 sp/s) stimulus with attention, relative to the ‘no attention’ condition [see right sight of Fig. 4(a), which shows the responses to the attended stimuli].

A larger modulation depth causes larger excitatory spike volleys in the populations of Poisson neurons and results in higher firing rates of the 41 HH-like neurons. Since the larger modulation depth impacts also the interneurons, this increased modulation can increase or even decrease the firing rate of the output neuron depending on the net balance between excitatory and inhibitory input. When both stimuli are presented simultaneously and when the preferred stimulus is attended, the effect of the larger excitatory spike volleys encoding the attended preferred stimulus is larger than the effect of inhibition by the increased firing rate of the interneuron. Therefore, the resulting firing rate $f_{\rm both}^{{\kern1pt}\rm att.pref}$ = 14.14 sp/s, SD = 0.05 sp/s [second line from top at the right side of Fig. 4(a)] is slightly larger than that in the condition of ‘no attention, both stimuli’ (*f*_both_ = 13.72 sp/s, SD = 0.05 sp/s). If the non-preferred stimulus is attended instead of the preferred stimulus, the effect of larger excitatory spike volleys is smaller than the effect of inhibition by the increased firing rate of the interneurons. Therefore, the resulting firing rate $f_{\rm both}^{{\kern1pt}\rm att.np} =$ 12.73 sp/s, SD = 0.05 sp/s [third line from top at the right side of Fig. 4(a)] is significantly lower than in the condition ‘no attention, both stimuli’.

In summary, the attended stimulus, presented alone, gives higher firing rates than the unattended stimulus alone due to the increased number of spikes in the population volleys. If both stimuli are presented and one is attended, the firing rate of the output neuron changes towards the firing rate elicited by that stimulus alone $\left( f_{\rm both}^{\rm att.np}<f_{\rm both}< f_{\rm both}^{\rm att.pref}\right)$.

Figure 4(b) shows that stimulus competition and the stimulus selection effect can also occur for other values of the modulation amplitude *A*_*m*_.

Obviously, the performance of the model depends on the strength of the excitatory projections of the non-preferred and preferred stimulus $(g_{\rm np}^{Y}$ and $g_{\rm p}^{Y})$, on their projections to the interneurons $(g_{\rm np}^{\rm int}$ and $g_{\rm p}^{\rm int})$, and on the synaptic connection *g*_inh_ of the interneurons to the output neuron. The results presented in Fig. 4(a) were obtained with a fixed set of parameter values. The results presented in Fig. 4(b) [with a modulation amplitude twice as large as in Fig. 4(a)] were obtained with the same parameter values except for *g*_inh_ which was decreased to 3.8 nS. The increase in modulation amplitude gives rise to an increased excitatory drive to both the output neuron *Y* and the inhibitory neuron. Since the output neuron is inhibited by the interneurons, the change in firing rate of the output neuron *Y* related to the increased modulation amplitude depends on the relative amounts of background noise, excitatory input and the strength of inhibition by the interneurons (Table 1). The new value for *g*_inh_ of 3.8 nS brings the firing rate of the output neuron to both stimuli halfway between that for the preferred and non-preferred stimulus only. Without reduction of *g*_inh_ the firing rate to both stimuli would have been strongly biased towards the firing rate for the non-preferred stimulus only (Result not shown).

**Table 1:** Average responses of an interneuron and the output neuron *Y*

*A*_*m*_	6 and 8		12 and 16	
Firing rate (Sp/s)	< *f*_*I*_ >	< *f*_*Y*_ >	< *f*_*I*_ >	< *f*_*Y*_ >
Non-pref only	0.28	8.09	0.90	11.43
Non-pref att	0.36	9.18	1.87	13.12
Pref only	0.002	14.15	0.01	19.81
Pref att	0.002	15.88	0.03	24.45
Both, non-pref att	12.85	12.73	19.38	14.47
Both	12.08	13.72	16.52	16.31
Both, pref att	12.36	14.14	18.04	16.86

In order to investigate to what extent the results in Fig. 4(b) depend on the particular choice of synaptic conductances, we have analyzed the model for a range of values of the relevant five synaptic conductances, $g_{\rm np}^{\rm int}$, $g_{\rm p}^{\rm int}$, $g_{\rm np}^{Y}$, $g_{\rm p}^{Y}$ and *g*_inh_. As it is difficult to visualize a five dimensional parameter space, we have varied the synaptic conductances of the non-preferred and preferred stimulus to the interneurons ($g_{\rm np}^{\rm int}$ and $g_{\rm p}^{\rm int}$), and tried to find the proper values for $g_{\rm np}^{Y}$, $g_{\rm p}^{Y}$ and *g*_inh_ such, that the model reproduced the properties of stimulus competition and selective attention. In detail, we adjusted the values of $g_{\rm np}^Y$, $g_{\rm p}^Y$ and *g*_inh_ for each pair of ($g_{\rm np}^{\rm int}$, $g_{\rm p}^{\rm int}$) values such that the model had the following properties:

1. the firing rate to the preferred and non-preferred stimulus alone should be in the range between 19.6 to 20.1 sp/s and 9.7 to 10.4 sp/s, respectively. As explained before this is implemented by the requirement that $g_{\rm p}^Y>g_{\rm np}^Y$;
2. the firing rate of the output neuron to the non-preferred and preferred stimulus presented together should be between the firing rates of the non-preferred and the preferred stimulus presented alone (stimulus competition);
3. attention should give higher firing rates than without attention, when the non-preferred or preferred stimulus is presented alone;
4. attention to either the non-preferred or preferred stimulus, presented simultaneously, changes the firing rate towards that for the attended non-preferred or preferred stimulus presented alone.

**Fig. 6:**
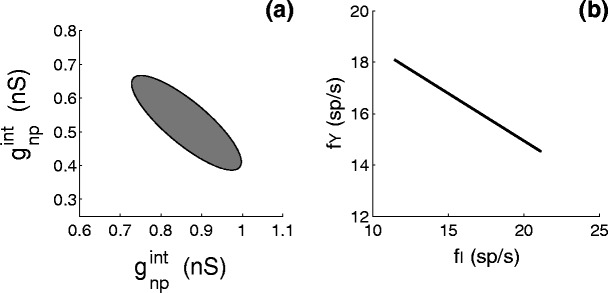
(**a**) Range of parameter values for $g_{\rm np}^{\rm int}$ and $g_{\rm p}^{\rm int}$ where the effects of attention, competition and selective attention can be reproduced. (**b**) Firing rates of the output neuron for the ‘no attention, both stimuli’ condition as a function of the corresponding average firing rate of the interneurons. Synaptic conductance values are chosen such that the effects of attention, competition and selective attention could be reproduced [*grey area* of (**a**)]

We found $1.45~nS \leq g_{\rm np}^{Y} \leq 1.52~nS$, $1.70~nS \leq g_{\rm p}^{Y} \leq 1.74~nS$ and 3.3 *nS* ≤ *g*_inh_ ≤ 5.04 *nS* for the three values of the synaptic connections to the output neuron *Y*, which are not shown in Fig. 6(a).

The fitted ellipse in Fig. 6(a) shows the range of parameter values for $g_{\rm np}^{\rm int}$ and $g_{\rm p}^{\rm int}$ where the effects of competition and selective attention can be reproduced for *A*_*m*_ = 12 (no attention) and *A*_*m*_ = 16 (with attention). For the region with parameters left of the grey area, either the inhibition is too small to reproduce the effect of stimulus competition or the inhibition is too strong, such that attention to the preferred stimulus does not increase but decrease the firing rate of the output neuron *Y*. For the region with parameters at the lower right of the grey area the model fails on a third aspect: attention to the non-preferred stimulus only decreases rather than increases the firing rate of the output neuron. Outside the upper boundary either the condition $f_{\rm both}^{\rm att.np} < f_{\rm both}$ or *f*_np_ < *f*_both_ is violated.

Figure 6(a) shows that stimulus competition and selective attention occur for different values of the pair ($g_{\rm np}^{\rm int}, g_{\rm p}^{\rm int}$). Variations in the parameter values $g_{\rm np}^{\rm int}$ and $g_{\rm p}^{\rm int}$ cause changes in firing rates of the interneurons. The fitted line in Fig. 6(b) shows the firing rate *f*_*Y*_ of the output neuron in the ‘no attention, both stimuli’ condition as a function of the firing rate *f*_*I*_ of the interneurons. The firing rate of the output neuron, when both the preferred and the non-preferred stimulus are presented falls between the firing rates of the preferred stimulus alone (20 sp/s) and of the non-preferred stimulus alone (10 sp/s). This firing rate is high (small) for low (high) firing rates of the inhibitory neurons.

In summary, our results show that i) the competition and attention effects as shown in Fig. 4 occur for a range of synaptic conductance values; ii) the firing rate in the ‘no attention, both stimuli’ condition takes values between *f*_np_ and *f*_p_.

### Simulation results for coherence estimate

**Fig. 7:**
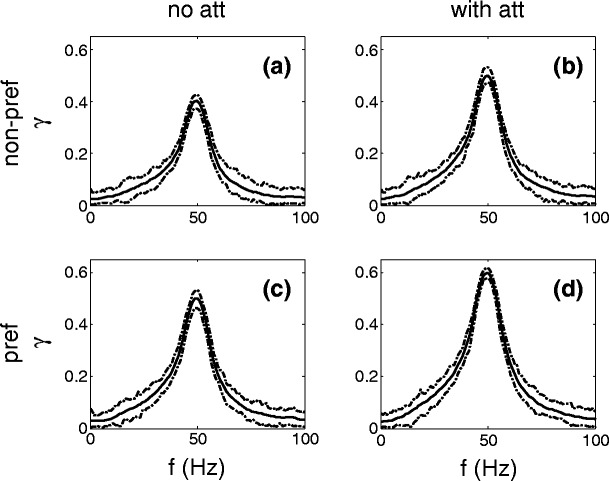
Coherence between the response and the modulation *A*_*m*_ *η*(*t*) of the non-preferred (*upper panels*) and preferred (*lower panels*) stimulus for the ‘no attention’ (*left panels*) and ‘with attention’ (*right panels*) condition in case just one stimulus is presented. The *dotted lines* show the 95% confidence level. Attention increases the peak value of the coherence estimate. The peak values of the coherence between the response and the non-preferred stimulus modulation are smaller than for the preferred stimulus

Figure 7 shows the coherence between the response of the output neuron and the time-dependent rate to populations *X*_1_ and *X*_2_ when either the non-preferred (upper row) or the preferred (lower row) stimulus is presented. The left and right column show the results for the ‘no attention’ (*A*_*m*_=6) and ‘with attention’ (*A*_*m*_=8) condition, respectively. Each of the panels shows a peak at 50 Hz, corresponding to the frequency content of the band-pass filtered stimuli.

Both for the non-preferred and preferred stimulus, the peak value of the coherence is larger for the ‘with attention’ condition (0.50 and 0.60, respectively) than for the ‘no attention’ condition (0.40 and 0.50, respectively). The 95% confidence level corresponds roughly to the range of the mean value, plus or minus 0.04. The larger coherence for the ‘with attention’ condition relative to the ‘no attention’ condition is due to the fact that larger spike volleys in the input will cause more precise spike timing (less variability). The peak values of the coherence for the non-preferred stimulus (Fig. 7(a, b)) are smaller than those for the preferred stimulus (Fig. 7(c, d)). This is caused by two facts: the preferred stimulus has stronger excitatory synapses to the output neuron than the non-preferred stimulus ($g_{\rm p}^Y>g_{\rm np}^Y$) and will therefore cause spikes which are more precisely time-locked to the stimulus. The second reason is that the stronger synaptic projections of the non-preferred stimulus to the interneurons cause more frequent inhibitory post-synaptic potentials in the output neuron, which can delay or even prevent the non-preferred stimulus to elicit a spike in the output neuron, resulting in a smaller coherence peak value.

**Fig. 8:**
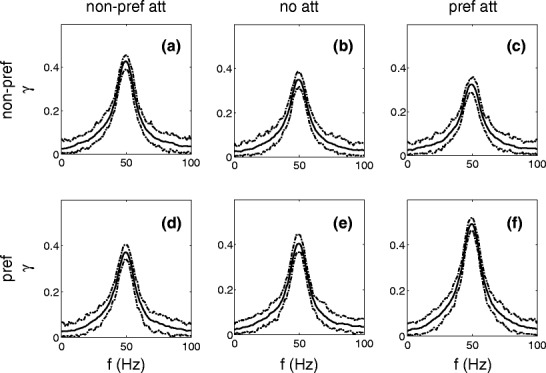
Coherence between the response and the modulation *A*_*m*_ *η*(*t*) of the non-preferred (*upper panels*) and of the preferred (*lower panels*) stimulus for different attention conditions. The *middle panels* (**b** and **e**) show the results when both stimuli are presented simultaneously and unattended. The *left panels* (**a** and **d**) show the coherence when the non-preferred stimulus has been attended, the *right panels* (**c** and **f**) when the preferred stimulus is attended. The 95% confidence level is shown by the *dotted lines*. Attention to one of the two stimuli results in a significantly larger peak value for the coherence for the attended stimulus and a significantly smaller peak value for the other stimulus

Figure 8 shows the coherence between the response of the output neuron and the input to population *X*_2_ (non-preferred stimulus) (upper row) and to population *X*_1_ (preferred stimulus) (lower row), respectively, when both stimuli are presented. The middle column [Fig. 8(b, e)] shows the results when both stimuli are presented simultaneously without attention (*A*_*m*_ = 6). For the non-preferred and preferred stimulus the coherence estimate has a peak value of 0.35 and 0.40, respectively. These two peak values are smaller than for the condition when these stimuli were presented alone [Fig. 7(a, c)]. When the non-preferred and preferred stimulus are presented simultaneously, the spikes of the output neuron reflect the contribution of both stimuli. The effect of the non-preferred (preferred) stimulus on the spike responses acts as a noise term in the response to the preferred (non-preferred) stimulus, which explains the smaller coherence values in Fig. 8 compared to that in Fig. 7.

When the preferred or non-preferred stimulus is attended [Fig. 8(a, f)], this stimulus becomes more effective, causing a better locking of the spike response to that stimulus. This more precise locking of the neuron to the attended stimulus leads to a larger coherence value for the attended stimulus and a lower coherence for the non-attended stimulus [compare Fig. 8(a, f) with Fig. 8(c, d), respectively]. The larger coherence for the attended preferred stimulus (0.49 vs. 0.40) and for the attended non-preferred stimulus (0.43 vs. 0.35) is significant (*p* < 0.001). The tendency that the coherence for the non-attended stimulus decreases when the other stimulus is attended (0.37 vs. 0.40 for the preferred and 0.33 vs. 0.35 for the non-preferred stimulus) is significant (*p* < 0.001). The 95% confidence level of the values corresponds roughly to the range of the mean value, plus or minus 0.04.

The coherence results for modulation amplitudes, which are twice as large, are similar and therefore not shown.

In summary: by attending a stimulus, the peak value of the coherence between the attended input and the response is larger compared to the condition ‘no attention’. The coherence between the non-attended input and the response does significantly decrease compared to the ‘both stimuli’ condition.

### Phase locking results

**Fig. 9:**
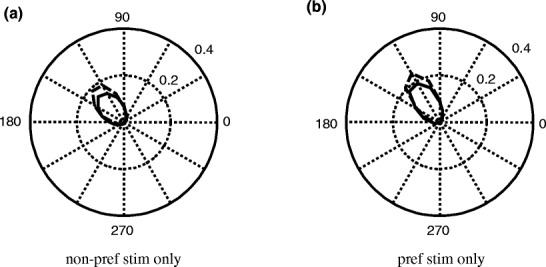
Polar plots of the fraction of phase differences between stimulus and response for the ‘one stimulus only’ condition. The *solid* (*dashed*)* line* shows the polar plots for the ‘no attention’ (‘with attention’) condition. (**a**) Shows the results for the ‘non-preferred stimulus only’ condition. (**b**) Shows the ratios for the ‘preferred stimulus only’ condition. The response is better phase locked to the preferred (**b**) than to the non-preferred stimulus (**a**); See text for further details)

Figure 9 shows polar plots of the probability distributions of phase differences between stimulus and response. The solid line shows the results for the ‘no attention’, the dashed line for the ‘with attention’ condition. For the ‘non-preferred stimulus only’ condition Fig. 9(a) shows that there is clear phase locking between the stimulus and the response of the output neuron which increases with attention (dashed line). The narrower the ellipse, the better the signals are locked to a certain phase difference and the higher the phase locking value (PLV) will be. The increase of the PLV for the ‘attention’ condition is significant (0.69 ± 0.01 (‘non-preferred stimulus only’) versus 0.80 ± 0.01 (‘attended non-preferred stimulus only’), *p* < 0.001), where phase locking values are given as the mean plus or minus the standard deviation. Figure 9(b) shows similar results for the ‘preferred stimulus only’ condition (mean PLV 0.80 ± 0.01 and 0.88 ± 0.01, *p* < 0.001, for the ‘non-attended preferred stimulus only’ and ‘attended preferred stimulus only’ condition, respectively).

**Fig. 10:**
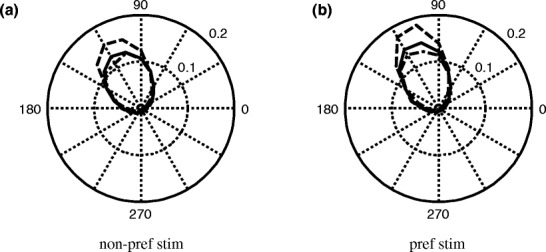
Polar plots for the fraction of phase differences between stimulus and response for the ‘no attention, both stimuli’ condition. (**a**) Shows the results for the non-preferred stimulus. Attending the non-preferred stimulus (*dashed line*) increases the phase locking between the non-preferred stimulus and the response compared to the ‘both stimuli with no attention’. Attending the preferred stimulus (*dashed-dotted line*) decreases the phase locking between the non-preferred stimulus and the response. (**b**) Shows the results for the preferred stimulus. Attending the preferred stimulus (*dashed line*) increases the phase locking between the preferred stimulus and the response compared to the ‘both stimuli with no attention’. Attending the non-preferred stimulus (*dashed-dotted line*) decreases the phase locking between the preferred stimulus and the response

Figure 10(a, b) show the polar distribution of the phase relation between input and spike output, when the preferred and non-preferred stimulus are presented simultaneously. Figure 10(a, b) shows the phase relation between the output and the input to *X*_2_ (non-preferred) and *X*_1_ (preferred), respectively. The solid line shows the results for the condition ‘no attention, both stimuli’, the dashed line for the condition ‘with attention’. Both panels show that attention increases the phase locking between input and response. The phase locking values for the non-preferred (preferred) stimulus are significantly larger for the condition ‘with attention’ (0.73 ± 0.01, 0.79 ± 0.01, respectively, *p* < 0.001) than for the ‘no attention, both stimuli’ (0.63 ± 0.01, 0.70 ± 0.01, respectively, *p* < 0.001) condition. The mean PLV for the preferred (non-preferred) stimulus for the ‘no attention, both stimuli’ condition, PLV = 0.70 ± 0.01 (0.63 ± 0.01 ) is significantly different for the condition ‘with attention to the other stimulus’, PLV = 0.64 ± 0.01 (0.59 ± 0.01).

The phase locking value results for two times larger modulation amplitudes are similar and therefore not shown.

In our simulations, the results of the coherence function and phase locking values at 50 Hz are very similar: attention significantly increases the coherence and the phase locking value between the response and the attended stimulus.

## Discussion

Many experimental and modelling studies have focussed on the neuronal implementation of attention (Bushnell et al. 1981; Spitzer et al. 1988; Motter 1993; McAdam and Maunsell 1999; Treue and Martinez-Trujillo 1999; Fries et al. 2001; Tiesinga 2005) and on stimulus competition (Moran and Desimone 1985; Desimone and Duncan 1995; Reynolds et al. 1999; Treue and Martinez-Trujillo 1999; Tiesinga 2005) at different levels of neuronal processing varying from brain areas (Corbetta and Shulman 2002) to single neurons (Deco and Rolls 2005; Tiesinga 2005). Most of these studies have focussed on firing rate to encode attended and unattended stimuli. However, it is well known that rhythmic neuronal activity, such as in *β*- and *γ*-oscillations, plays an important role in encoding sensory stimuli (see e.g. Kreiter and Singer 1996) and that attention affects the amplitude of the rhythmic neuronal oscillations. The latter is illustrated by the coherence between the local field potential and spike output, which provides a sensitive measure of local neuronal synchronization. Fries et al. (2001) found that for the ‘with attention’ condition, the coherence between the local field potential and the simultaneously recorded spike train was significantly larger with than without attention to the stimulus. Our results will be discussed in more detail below, starting with a comparison of the model responses with other models.

The architecture of our model is quite similar to the gain modulation model by Reynolds and coworkers (Reynolds et al. 1999). The main differences with respect to the gain modulation model are related to the nature of the neuronal input signals and to the neuronal implementation of attention. In the Reynolds model constant firing rates are used to encode the preferred and non-preferred stimuli and attention was implemented by a five-fold increase of the efficacy of the synapses that transmit the attended stimulus. This model left open the question of how synaptic efficacy can be modulated selectively for the attended stimulus input at such a short time scale. In agreement with experimental observations (Fries et al. 2001), our working hypothesis was that attention is implemented through enhanced gamma activity, which makes this input more effective in eliciting a spike in the output neuron, and thus increases the effective strength of the signal encoding the attended stimulus.

Our model is an alternative for the model proposed by  Tiesinga (2005) which postulates a stimulus-related excitatory input without rhythmic oscillations and with top–down input from the frontal eye fields (FEF). The main difference between our model and that by  Tiesinga (2005) is that we assume that attention is implemented in the *γ*-modulated stimulus-related neural input, whereas  Tiesinga (2005) does not assume *γ*-modulated stimulus-related input. In the Tiesinga model the *γ*-oscillations are postulated to be induced by FEF input to the interneurons. Although it is well know that the FEF is involved in attention-related modulations of neuronal activity  (Moore and Amstrong 2003), it is still a matter of debate how the FEF input affects the neuronal processing. Our model allows a role for top–down attentional modulation of the amplitude of the *γ*-oscillations in the input population activity representing the visual stimulus. It is a topic for future research to investigate the details of attention-related top–down mechanisms.

One of the values of models is that they can provide possible explanations for experimentally observed phenomena. When developing a model, one should always try to explain as many experimental findings with as few as possible model assumptions. In our model, we assumed that the stimulus-related neuronal activity has rhythmic oscillatory components. This assumption is supported by experimental observations which have revealed stimulus-related rhythmic activity in V1 (van der Togt et al. 2006; Roelfsema et al. 2004; Rols et al. 2001), V2 (Frien et al. 1994) and V4 (Fries et al. 2001; Taylor et al. 2005). Moreover, we assume that attention is implemented by increased amplitudes of the rhythmic excitatory activity. This is in agreement with experimental observations by Fries et al. (2001), Taylor et al. (2005), Womelsdorf et al. (2006), who reported that attention is related to an increased coherence between local field potentials and single-unit activity. The simple feed-forward model reproduces experimental data of stimulus competition and attention effects on firing rate (see e.g. Reynolds et al. (1999)). Moreover, our model predicts an increased peak value of the coherence due to attention, emphasizing the increased neuronal synchronization by attention. Our predictions concerning an increased coherence for attended stimuli and a decreased coherence in case the other stimulus within the receptive field is attended, are in agreement with what is found by Smiyukha et al. (2006). These authors placed two small stimuli close to each other, causing two spatially well separated foci of gamma-band activity in area V1 of a macaque. The corresponding foci in V4 were largely overlapping. Wavelet based analysis of correlations revealed strong synchronization of field potentials in the gamma-band between the site in V1, processing the attended shape, and the site in V4 responsive to both stimuli. Synchronization with activity in V4 is weak for other sites in V1, processing non-attended stimuli. This strong synchronization between the area in V1, which processes the attended stimulus, and the site in V4, is at least qualitatively similar to the increase in coherence between input and spike output in our model.

Recently, a model with an architecture very similar to our model was proposed by Mishra et al. (2006) to explain the phenomena of stimulus competition and selective attention. The neuronal mechanisms in their model to explain stimulus competition are feedforward inhibition, like in our model, and synaptic depression, which is effective for input frequencies of 40 Hz and above. Like in our model, each stimulus is represented by excitatory multi-unit activity. In their model the excitatory neuronal signals that encode the preferred and non-preferred stimuli are always in anti-phase. The phenomenon of selective attention in their model is achieved by imposing a phase shift of the response of the interneuron relative to the excitatory activity encoding the attended stimulus. This implies that the inhibition is more or less in anti-phase with the excitatory drive of the attended stimulus, but in phase with the excitatory drive of the unattended stimulus. Therefore, the excitatory input of the unattended stimulus is cancelled by inhibition from the interneuron. This works well when the excitatory drive and the inhibitory input from the interneurons is tuned at the same frequency (40 Hz in the paper by Mishra et al. (2006)) and more or less in anti-phase. However, experimental studies have found that rhythmic synchronization is broadly tuned and that the neuronal activity representing two different stimuli is uncorrelated (Gray et al. 1989; Kreiter and Singer 1996). Therefore, we decided to generate the neuronal signals for attended and unattended stimuli by band-pass filtering two independent noise signals.

Fries et al. (2001) showed that the amplitude of the input fluctuations of neurons in V4 is larger when the stimulus is attended than when the same stimulus is not attended. Therefore, we increased the amplitude of our input modulation by 33% to implement the effect of attention (from *A*_*m*_ = 6 to 8). This increase in amplitude caused a 12% higher firing rate of the output neuron, a 20–25% increase in the coherence between input and output and a 10–16% larger phase locking value (PLV). We have also done the simulations for amplitudes of the input modulations, twice as large. Now, the 33% increase in amplitude of the input modulation, which implemented the effect of attention, caused a 15–25% higher firing rate of the output neuron, a 11% increase in the coherence between input and output and a 3% larger PLV. This indicates that all results for firing rate, coherence and phase coherence are qualitatively similar, independent of modulation amplitude, showing that our model is quite robust.

The results of this study were obtained for various modulation amplitudes of the stimulus-related input with the same set of parameters, except for the value of *g*_inh_ which was reduced from 4.5 to 3.8 nS when the modulation amplitude was made twice as large. If we had kept the synaptic strength at 4.5 nS, the response of the output neuron to both stimuli would have been more biased towards the output for the non-preferred stimulus. As far as we know there have been no studies which have systematically investigated the effect of changes in modulation amplitude of excitatory drive to neurons in V2 and V4 on stimulus competition. Maybe a bias to the response to the non-preferred stimulus alone for larger modulation amplitudes is what will be observed. Another alternative might be that dynamic synapses (Tsodyks et al. 1998, 2000) reduce the effective synaptic strength of the projection of the inhibitory neurons to the output neuron when the increased modulation amplitude causes a larger increase of the firing rate of the inhibitory neurons. The latter seems a plausible mechanism which we saw as a justification to reduce the synaptic efficacy of the projections of the inhibitory neurons. The size of the reduction is certainly not critical to qualitatively reproduce the results in this study.

A similar robustness was found for variations in the synaptic strengths. As shown in Fig. 6(a) stimulus competition could be reproduced over a range from 0.7 to 1.0 nS for $g_{\rm np}^{\rm int}$ and from 0.4 to 0.7 nS for $g_{\rm p}^{\rm int}$. Changes in the parameter values lead to variations in the firing rate of the output neuron [Fig. 6(b)]. For the condition ‘no attention, both stimuli’ this range goes from about 15 sp/s to 18 sp/s depending on the firing rates of the inhibitory neurons. This range of attenuation of the firing rate of the output neuron to both stimuli compared to the firing rate for the preferred stimulus alone, is within the range reported by Reynolds et al. (1999) and Gawne and Martin (2002) for V4, by Miller et al. (1993) for the inferior temporal cortex and by Rolls and Tovee (1995) in the anterior part of the superior temporal sulcus.

We want to remark that the effect of competition in the experimental results in the literature is not always as large as in the paper shown by Reynolds et al. (1999). See for example the study of Gawne and Martin (2002). For a substantial fraction of the neurons these authors found that the firing rate to both stimuli was close to the highest firing rate to the stimuli presented separately.

**Fig. 11:**
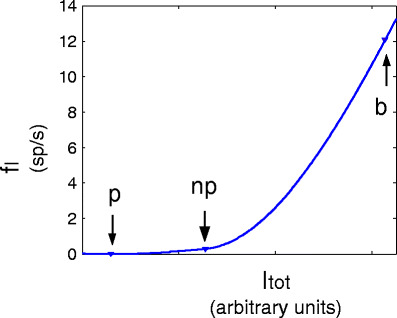
Firing rate *f*_*I*_ of the interneurons as a function of the total synaptic input current *I*_*tot*_ to the interneuron. The *three arrows* refer to the mean current input to the inhibitory neurons for the different conditions: ‘preferred stimulus only’ (*p*), ‘non-preferred stimulus only’ (*np*) and ‘both stimuli’ (*b*)

Lachaux et al. (1999) showed that the coherence cannot distinguish phase and amplitude covariance. As an alternative they introduced the PLV to detect phase synchrony. For our simulation results we determined the coherence values (typically 0.33–0.60) as well as the phase locking values (typically 0.59–0.88). These two sets of values lead to the same qualitative conclusions: (1) the input and response of the output neuron *Y* are more synchronized if the stimulus, represented by the neuronal input, is attended compared to be not attended; (2) the input representing the not-attended stimulus is less synchronized with the response of the output neuron *Y* than the input which represents the simultaneously offered but attended stimulus. In addition, the polar plots of Figs. 9 and 10 show that the average phase difference between input and response of the output neuron *Y* are different for the stimulus conditions ‘non-preferred stimulus, only’, ‘preferred stimulus, only’ and ‘both stimuli’. The average phase difference for the ‘preferred stimulus, only’ condition [< *ϕ*_*p*_ > ~ 125°, Fig. 9(b)] is smaller than for the ‘non-preferred stimulus, only’ condition [< *ϕ*_np_ > ~ 133°, Fig. 9(a)]. This can be explained by the fact that the maximum conductance of the excitatory synapses to the output neuron is larger for the excitatory neuronal activity representing the preferred than for that representing the non-preferred stimulus. For the condition ‘both stimuli, no attention’ this average phase difference [< *ϕ*_both_ > ~ 114°, solid line in Fig. 10(a, b)] is even smaller since the neuron receives more input, so that it can generate even faster a spike (in case it is not inhibited! The input-output relation for the output neuron without inhibition is comparable to that for the inhibitory neurons shown in Fig. 11).

In this study we have presented a feedforward model which can reproduce neuronal responses in visual cortex related to stimulus competition and selective attention effect, by: (1) using gamma-modulated population activities to represent the stimuli; (2) increasing the modulation depth of the population activity representing the attended stimulus; (3) determining the non-preferred and preferred stimulus response by using different values for each group of the various synaptical conductances of the interneuron and output neuron. Our numerically obtained firing-rate results are similar to experimental results reported by Reynolds et al. (1999), Miller et al. (1993) and Rolls and Tovee (1995).

## Appendix 1

The parameter values for the voltage-dependent Na^ + ^ and K^ + ^ currents were described by (Traub and Miles 1991):

5 $$ I_{\rm Na} = \bar{g}_{\rm Na} m^3 h (V-E_{\rm Na}) $$

6 $$ \frac{dm}{dt} = \alpha_m(V)(1-m)-\beta_m(V)m $$

7 $$ \frac{dh}{dt} = \alpha_h(V)(1-h)-\beta_h(V)h $$

8 $$ \alpha_m = \frac{-0.32(V-V_T-13)}{\exp[-(V-V_T-13)/4]-1} $$

9 $$ \beta_m = \frac{0.28(V-V_T-40)}{\exp[(V-V_T-40)/5]-1} $$

10 $$ \alpha_h = 0.128 \exp[-(V-V_T-V_S-17)/18] $$

11 $$\beta_h = \frac{4}{1+\exp[-(V-V_T-V_S-40)/5]}$$

where *V*_*T*_ = − 58 mV and *V*_*S*_ = − 10 mV (Destexhe and Paré 1999). $\bar{g}$ is the maximum conductance ($\bar{g}_{\rm Na} = 361.2\, 10^{-4}$ S/cm^2^, $\bar{g}_{Kd} = 70\, 10^{-4}$ S/cm^2^), *m*, *h* and *n* are the time-varying gate variables, *E*_Na_ = 50 mV is the sodium reversal potential, *E*_*Kd*_ = − 90 mV the potassium reversal potential, *α* is the forward and *β* the backward rate. The “delayed-rectifier” K^ + ^ current was described by:

12 $$ I_{Kd} = \bar{g}_{Kd} n^4(V-E_K) $$

13 $$ \frac{dn}{dt} = \alpha_n(V)(1-n)-\beta_n(V)n $$

14 $$ \alpha_n = \frac{-0.032(V-V_T-15)}{\exp[-(V-V_T-15)/5]-1} $$

15 $$ \beta_n = 0.5 \exp[-(V-V_T-10)/40] $$

In our model both the interneurons and the output neuron receive background synaptic input as received by cortical neurons *in vivo*, represented by fluctuating background conductance injections in the soma. These conductances are produced by an Ornstein–Uhlenbeck process, as described by Destexhe et al. (2001):

16 $$ \frac{dg(t)}{dt} = \frac{g_0-g(t)}{\tau}+\chi(t)\sqrt{\frac{\sigma^2}{\tau}} $$

where *g*_0_ is the mean conductance, *τ* is the conductance time constant, *σ* ^2^ is the variance of the conductance and *χ*(*t*) is Gaussian white noise with zero mean and a standard deviation of 1. For the inhibitory background conductance of the output neuron we use *g*_*i*0_ = 57.3 nS, *τ*_*i*_ = 10.49 ms and *σ*_*i*_ = 6.0 nS with a reversal potential *E*_*i*_  = − 75 mV. For the excitatory background conductance of the output neuron we use *g*_*e*0_ = 12.1 nS, *τ*_*e*_ = 2.73 ms and *σ*_*e*_ = 3.0 nS with a reversal potential *E*_*e*_  =  0 mV.

For the interneurons the average conductances and the standard deviations of these conductances are 50% of the corresponding values of the output neuron.

For the implementation in NEURON of the passive and active properties and of the synaptic background we used parts of the code of example 5 of the NEURON tutorial from the Obidos 2004 course http ://www.neuron.yale.edu/ftp/neuron/contrib/obidos_tutorials/.

## Appendix 2

In this appendix we provide a rough estimate of the currents injected as a noisy background and the currents due to the stimulus related inputs to the conductance based neurons in our model. Current and conductance are related by:

17 $$ I_{\rm i,e}(t) = G_{\rm i,e}(t) (V(t) - E_{\rm i,e}) $$

where *G*_i,e_ is the total conductance, *E*_i,e_ the reversal potential and *V* the membrane potential for the inhibitory (i) or excitatory (e) input. The total input current is the sum over all excitatory and inhibitory currents. The amount of current is time-dependent since it is a function of the fluctuating membrane potential and of the total amount of the conductance at time t. We will approximate the currents by taking time-averages for conductance and membrane potential. The mean value for the membrane potential *V* depends on the contribution of all excitatory and inhibitory inputs. From the simulations in NEURON we know that the average membrane potential < *V* > of the interneurons (output neuron *Y*) is about − 55 mV (− 60 mV, respectively), i.e., well between the rest membrane potential near − 75 mV and the threshold for action potential generation. The value of the reversal potentials are *E*_*i*_ = − 75 mV and *E*_*e*_ = 0 mV for both the interneuron and excitatory neuron, with these values the average currents caused by the noisy background in the interneurons is

18 $$\begin{array}{*{20}l} I_i = & g_{i0}^I \left(<V^I{\kern-1.5pt}>-{\kern-1.5pt}E_i\right) & {\kern-1.5pt}={\kern-1.5pt} ~-0.6 ~n{\kern-1.5pt}A\nonumber\\ I_e = & g_{e0}^I \left(<V^I{\kern-1.5pt}>{\kern-1.5pt}-E_e\right) & {\kern-1.5pt}={\kern-1.5pt} ~0.3~n{\kern-1.5pt}A \end{array}$$

The average input currents caused by the stimuli in the interneurons is

19 $$\begin{array}{*{20}l} I_i = & N_i~{\kern-1.5pt}<{\kern-1.5pt}f_{I}>~g_{\rm inh}~\tau_i \left(<V^I{\kern-1.5pt}>-{\kern-1.5pt}E_i\right) & =\; -0~nA \nonumber\\ I_e = & N_e~{\kern-1.5pt}<{\kern-1.5pt}r>~\left(g_p^{\rm int} {\kern-1.5pt}+{\kern-1.5pt}~g_{\rm np}^{\rm int}\right) \tau_e \left(<V^I>-E_e\right) {\kern-1.5pt} & ={\kern-1.5pt} ~0.2~nA \nonumber\\ \end{array}$$

with *N*_i,e_ the number of input spike trains, < *f*_*I*_ > the average firing rate of the stimulus-related inhibitory input spike trains, < *r* > the average rate of the stimulus-related excitatory Poisson spike trains and *τ*_i,e_ the rise time of the *α*-synapses. This means that the inhibitory input current of the interneurons is only due to the noisy background and the excitatory current comes for 58% from the noisy background and 42% comes from the stimulus related input.

The average input currents caused by the noisy background in the output neuron are

20 $$\begin{array}{*{20}l} I_i & = g_{i0} \left(<V^Y>-E_i\right) & = ~-0.9~nA \nonumber\\ I_e & = g_{e0} \left(<V^Y>-E_e\right) & = ~0.7~nA \end{array}$$

where *g*_0_ is the mean conductance of the conductance injections in the soma as described in Appendix 1. The average input currents caused by the stimuli in output neuron *Y* are

21 $$\begin{array}{*{20}l} I_i & =& N_i~{\kern-1.5pt}<{\kern-1.5pt}f_{I}{\kern-1.5pt}>{\kern-1.5pt}~g_{\rm inh}~\tau_i \left({\kern-1.5pt}<{\kern-1.5pt}V^Y{\kern-1.5pt}>{\kern-1.5pt}-E_i\right) {\kern-1.5pt}={\kern-1.5pt}-\,0.2~nA \nonumber\\ I_e & =& N_e~{\kern-1.5pt}<{\kern-1.5pt}r>~\left(g_p^Y~{\kern-1.5pt}+{\kern-1.5pt}~g_{\rm np}^Y\right)~\tau_e~\left({\kern-1.5pt}<{\kern-1.5pt}V^Y{\kern-1.5pt}>{\kern-1.5pt}-E_e\right) {\kern-1.5pt}={\kern-1.5pt} ~~0.6~nA \nonumber\\ \end{array}$$

with *N*_i,e_ the number of input spike trains, < *f*_*I*_ > the averaged inhibitory input spike train, < *r* > the averaged rate of the excitatory Poisson spike train, *τ*_i,e_ the rise time of the *α*-synapses. The noisy background is responsible for 84% of the total inhibitory input current and the stimulus related input contributes 16%. The excitatory current contributes for 54% of the noisy background and 46% is due to the stimulus related input.

The input current for the ‘preferred stimulus only’ condition (p) consist of the background current plus the excitatory related to the preferred stimulus. For the ‘non-preferred stimulus only’ condition (np) the excitatory stimulus-related current to the inhibitory neuron is larger than the current for the preferred stimulus because of the larger value of the synaptic connections for the non-preferred stimulus input.
